# Supplementary material for: In situ architecture of the lipid transport protein VPS13C at ER–lysosome membrane contacts
Source: Proc Natl Acad Sci U S A. 2022 Jul 13;119(29):e2203769119. doi: 10.1073/pnas.2203769119 (PMC9303930; doi:10.1073/pnas.2203769119)
Supplement: Supplementary File [file pnas.2203769119.sapp.pdf]

Supplementary Information for

In situ architecture of the lipid transport protein VPS13C at ER-lysosomes membrane contacts

Shujun Cai[1,2,3,4,5], Yumei Wu[1,2,3,4,5], Andrés Guillén-Samander [1,2,3,4,5], William Hancock-Cerutti[1,2,3,4,5], Jun Liu[2*] and Pietro De Camilli[1,2,3,4,5*]

[1]Department of Neuroscience, Yale University School of Medicine, New Haven, CT 06510, USA;
[2]Department of Cell Biology, Yale University School of Medicine, New Haven, CT 06510, USA;
[3]Howard Hughes Medical Institute, Yale University School of Medicine, New Haven, CT 06510, USA;
[4]Program in Cellular Neuroscience, Neurodegeneration and Repair, Yale University School of Medicine, New Haven, CT 06510, USA;
[5]Aligning Science Across Parkinson's (ASAP) Collaborative Research Network, Chevy Chase, MD 20815, USA.

* Correspondence:
Pietro De Camilli: pietro.decamilli@yale.edu
Jun Liu: jliu@yale.edu

This PDF file includes:

Figs. S1 to S7

Captions for movies S1 to S6

Tables S1 to S2

References for SI citations

Other supplementary materials for this manuscript include the following:

Movies S1 to S6

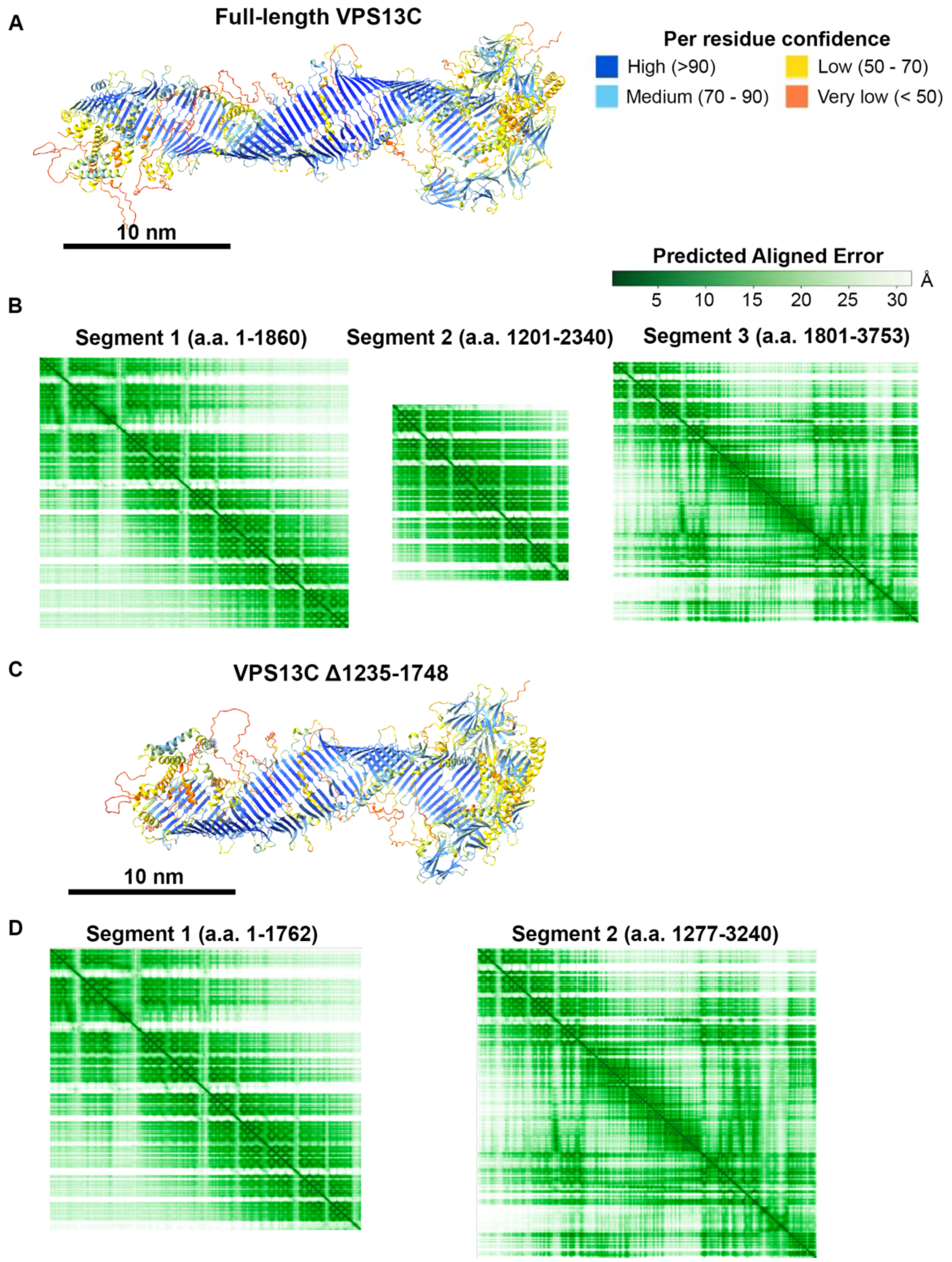

Figure S1. Full-length and truncated VPS13C structure colored by confidence score per residue pLDDT.

(A) Predicted structure of full-length VPS13C colored by confidence score per residue pLDDT. The global pLDDT of three segments, a.a. 1-1860, 1201-2340, 1801-3753, are 73.4, 78.5, 74.4, respectively (B) Plots colored by the pairwise PAE values for three predicted segments of full-length VPS13C (C) Predicted structure of VPS13C Δ1235-1748 colored by confidence score per residue pLDDT. The global pLDDT of two segments, a.a. 1-1762 and 1277-3240, are 72.3 and 74.7, respectively (D) Plots colored by the pairwise PAE values for two segments of VPS13C Δ1235-1748.

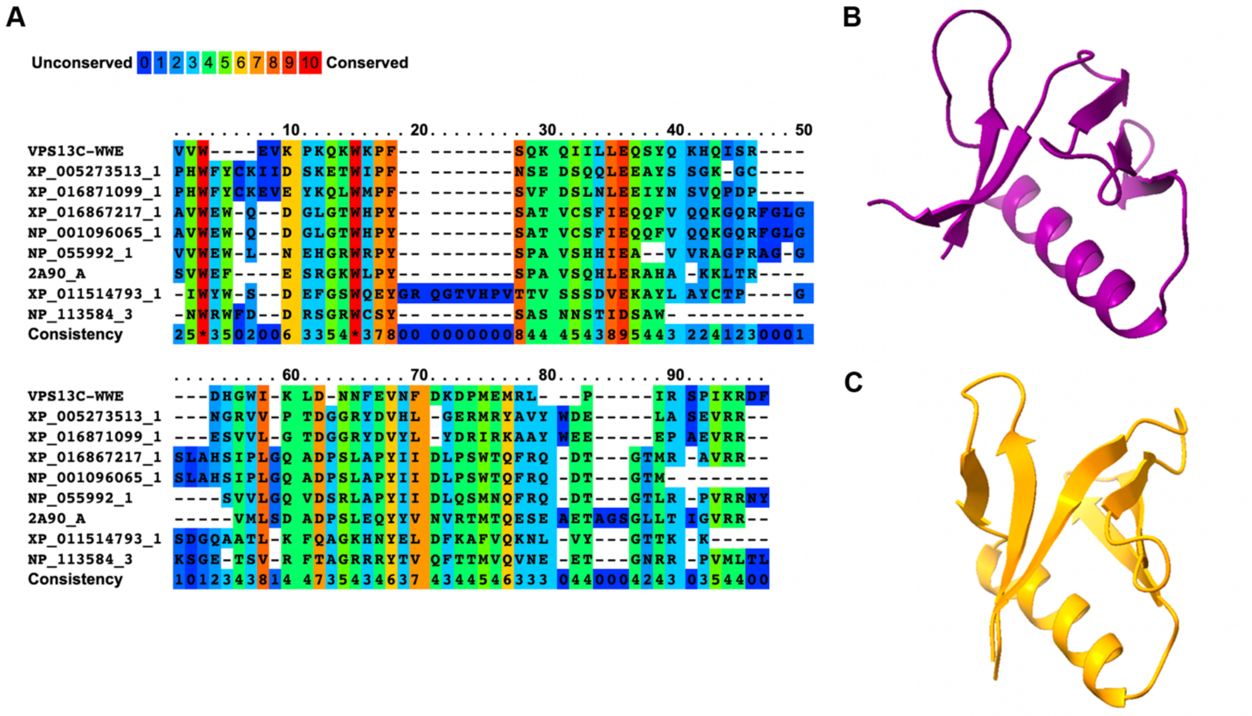

Figure S2. A fragment of VPS13C (a.a 3117-3183) aligns with WWE domains.

(A) Sequence alignment of a VPS13C fragment (a.a 3117-3183) with WWE domains of multiple proteins by PRALINE multiple sequence alignment[1]. (B) Structural prediction of VPS13C3117-3183 by AlphaFold v2.0 (C) Structure of WWE domain of human HUWE1 (PDB 6MIW).

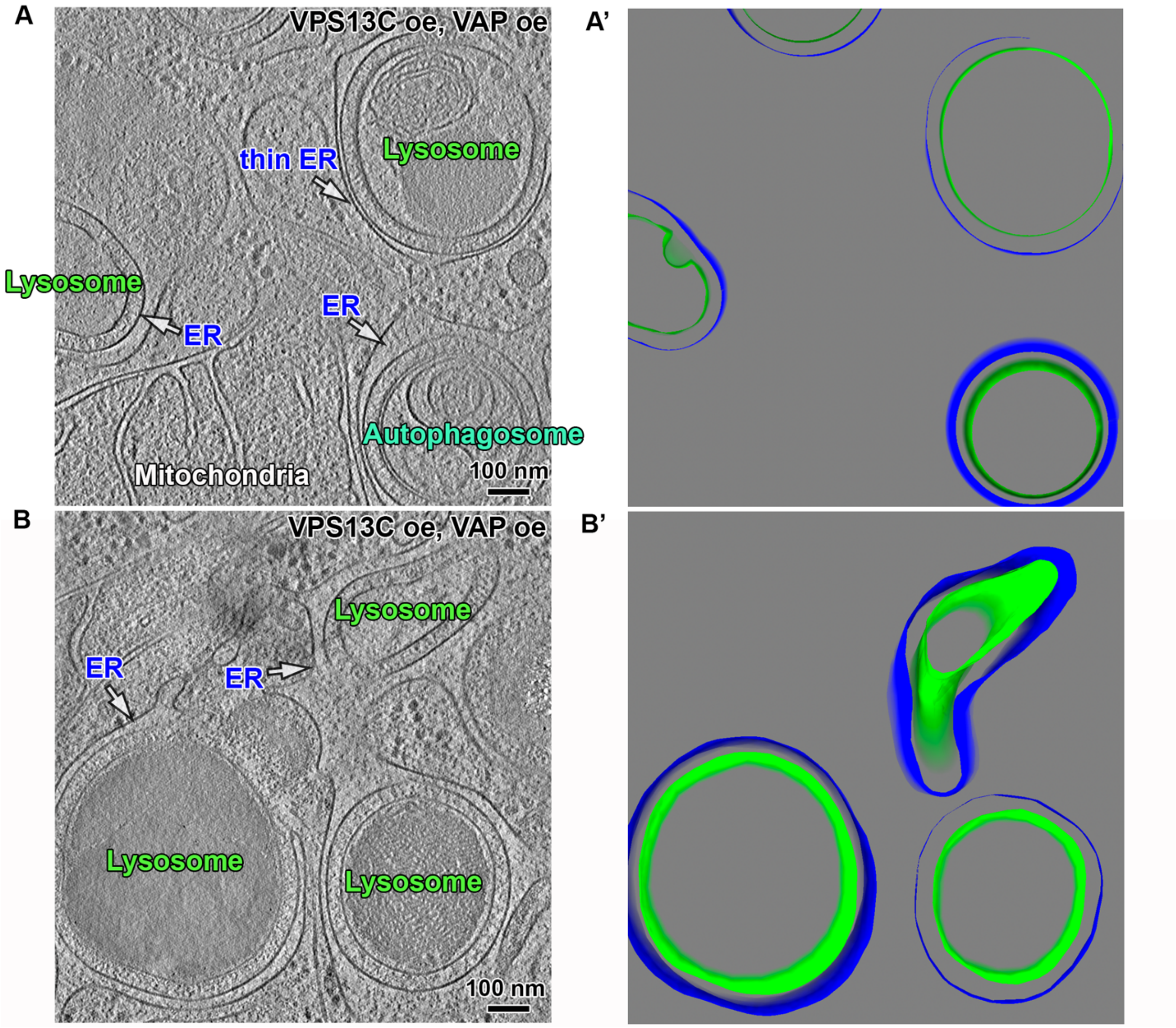

Figure S3. Examples of VPS13C mediated contact sites.

(A and B) Cryotomographic slices (1-nm thick) showing VPS13C-mediated membrane contacts in HeLa cells overexpressing VPS13C^Halo and GFP-VAP-B. (A' and B') 3D segmentation views of panels A and B, respectively, showing endo/lysosome membranes in green, autophagosome double membranes in cyan and ER membranes in blue. Intermembranes spaces are partially hidden by the 3D representation.

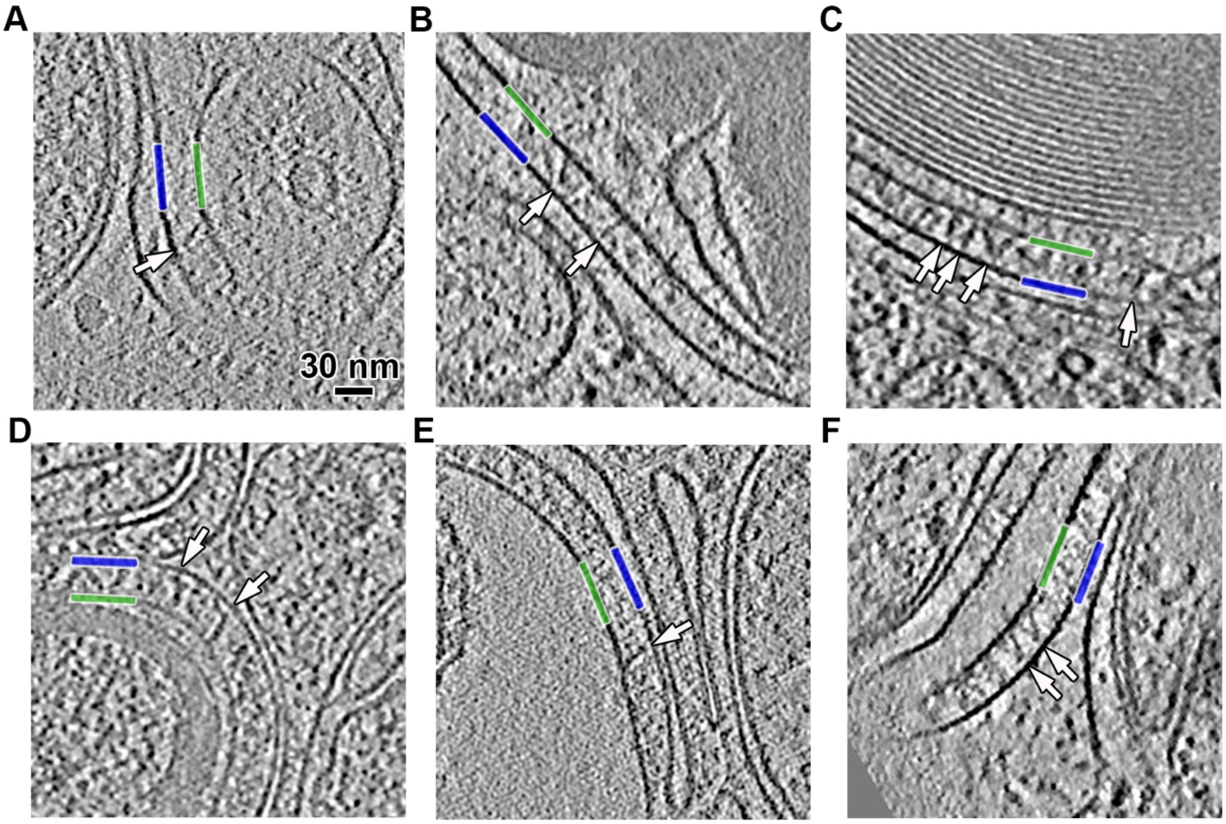

Figure S4. Example tomographic slices showing rod-shaped densities bridging ER-endo/lysosome.

(A-E) Cryotomographic slices (1-nm thick) showing rod-shaped densities at ER-lysosome contacts in HeLa cells overexpressing full-length VPS13C and VAP. Blue and green lines: ER and lysosome membrane, respectively. Arrows point to rod-shaped densities bridging ER and lysosome membranes.

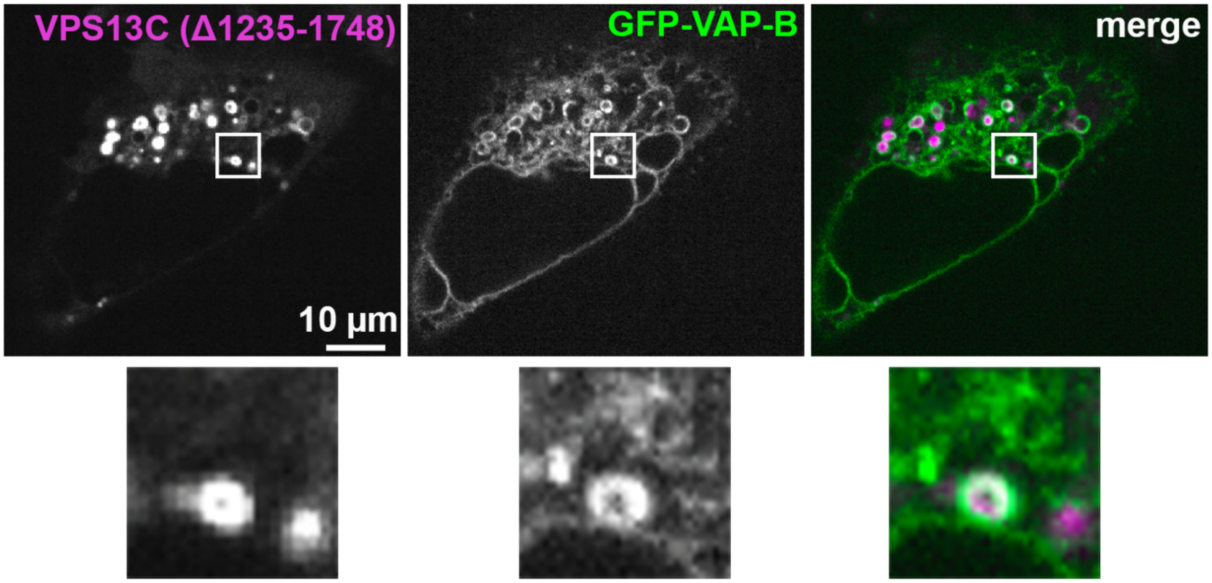

Figure S5. The VPS13C truncation mutant (Δ1235-1748) has the same subcellular localization as full-length VPS13C.

Confocal microscopy z-slice images of a HeLa cell expressing VPS13C^Halo (Δ1235-1748) and GFP-VAP-B in the split and merged channels. The region enclosed by a box is shown at five fold enlargement at the bottom.

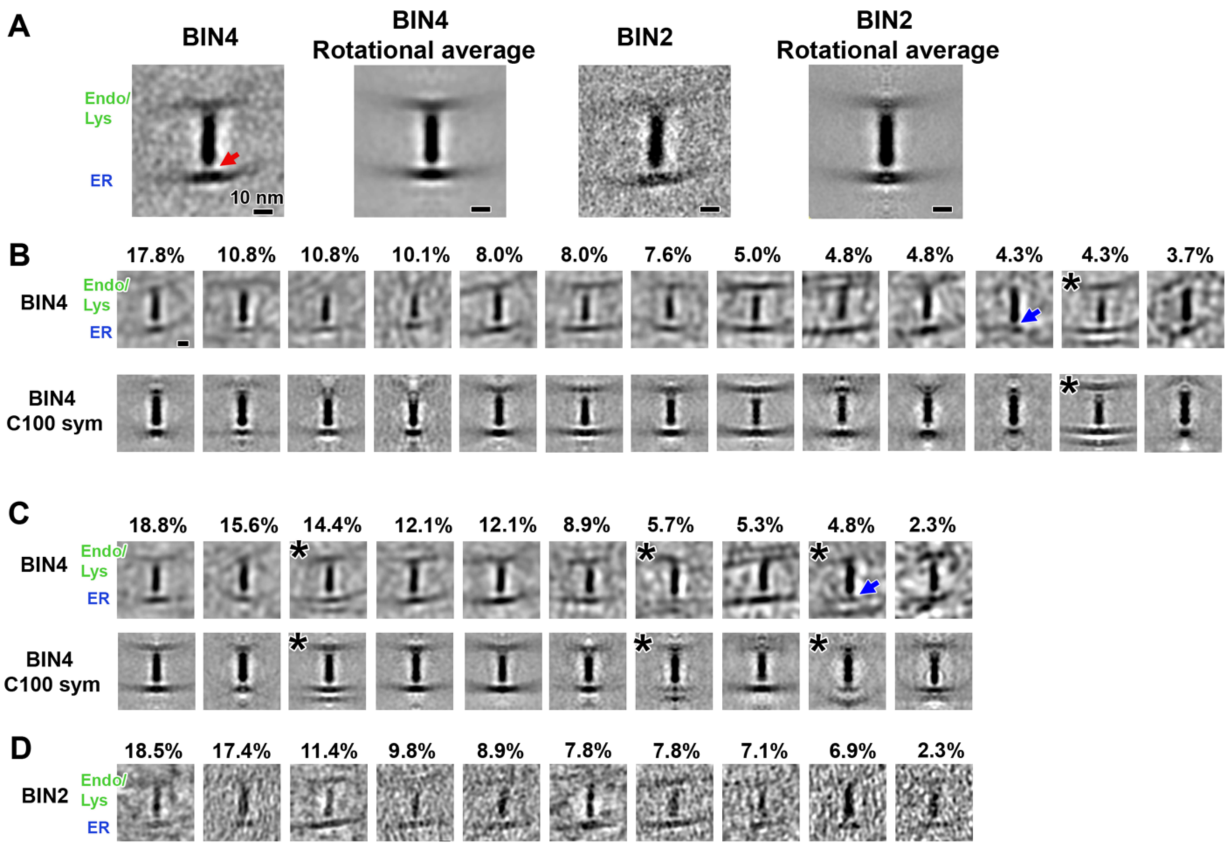

Figure S6. Subtomogram averaging and 3D classification.

(A) Subtomogram averages with subtomograms binned by 4 and binned by 2, respectively. Red arrow points to a gap between the N-terminal end of the VPS13C rod and the ER membrane.
(B) Class averages (3D) of subtomograms binned by 4. Blue arrow points to a class showing no obvious gap between the N-terminal end of the VPS13C rod and the ER membrane. In the fields labeled by an asterisk the two membranes visible at the bottom represent the two membranes of the thin ER.
(C) Class averages (3D) of subtomograms binned by 4. A mask around ER side of the rod was applied. Blue arrow points to a class showing no obvious gap between the N-terminal end of the VPS13C rod and the ER membrane. In the fields labeled by an asterisk the two membranes visible at the bottom represent the two membranes of the thin ER.
(D) Class averages (3D) of subtomograms binned by 2.

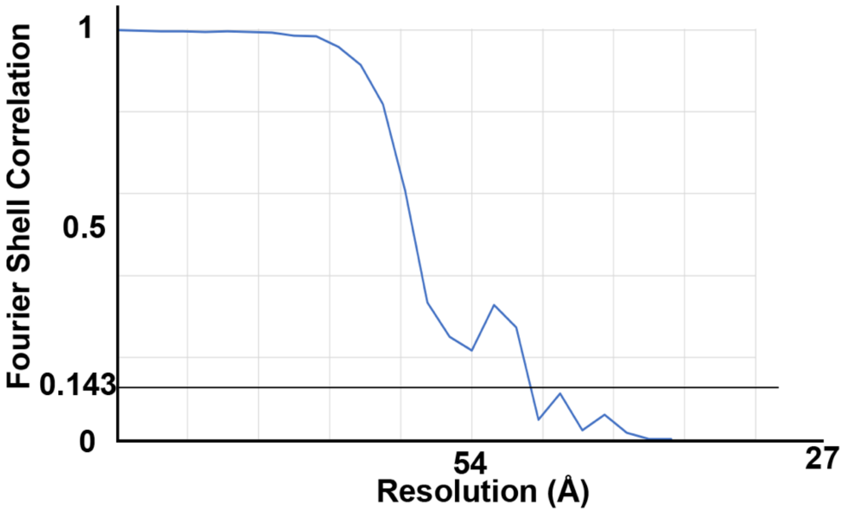

Figure S7. Fourier Shell Correlation of the structure shown in Figure 4F.

The resolution of the averaged density map in Figure 4F is ~ 47 Å, based on the Fourier Shell Correlation = 0.143 criterion.

Movie S1. Predicted structure of full-length VPS13C bound to the MSP domain of VAP.

The color scheme is the same as the one shown in Fig 1A.

Movie S2. Predicted structure of full-length VPS13C showing a continuous hydrophobic groove

Surface representations of the structure of VPS13C predicted from AlphaFold showing carbon atoms in grey (hydrophobic surfaces), oxygens in red (negative charges), nitrogens in blue (positive charges) and sulfur in yellow. For clarity of presentation, some α-helices and disordered loops are not shown.

Movie S3. FIB-SEM image stack of endo/lysosomes from Cos-7 cells overexpressing VPS13C^halo and PDZD8-EGFP

FIB-SEM image stack of the structure shown in Fig. 2E'. VPS13C and PDZD8 segregate to distinct domains within the contact area between ER (thin ER) and the endo/lysosome.

Movie S4. Cryotomogram of a HeLa cell overexpressing full-length VPS13C and VAP

This cryotomogram shows abundant ~30-nm rod-shaped bridging densities at ER-lysosome contacts.

Movie S5. Cryotomogram of a HeLa cell overexpressing a truncated VPS13C mutant (VPS13C Δ1235-1748) and VAP.

This cryotomogram shows shorter rod-shaped densities and narrower intermembrane space at ER-lysosome contacts, compared to the ones mediated by full-length VPS13C.

Movie S6. Three-dimensional view of VPS13C averaged density map

Three-dimensional view of the VPS13C rod bridging two membranes. The density corresponding to VPS13C is shown in light orange, fitted with the full-length VPS13C predicted structure from AlphaFold v2.0. The color scheme of predicted structure is the same as that of Fig. 1A. The densities corresponding to ER and endo/lysosome membrane are colored in blue and green, respectively.

Table S1. Cryo-ET and image-analysis details.

EM grid	Quantifoil R 2/4, LF, 200 Mesh, Gold
Microscope	Titan Krios
Energy	300 kV
Camera: recording mode	Gatan K3 Summit: counting
Subframes / tilt	10
Energy filter / zero-loss slit width	GIF energy filter/ 20 eV
Tomography software	SerialEM
Calibrated pixel size, unbinned	3.3 Å
Defocus (nominal, defocus contrast)	-6 µm
Defocus (nominal, Volta contrast)	-1 µm
Cumulative dose	80 e- / Å2
Dose fractionation	constant
Tilt range	±50-60°, dose-symmetric
Tilt increment	3°
Movie alignment	MotionCor2
Tomogram processing & visualization	IMOD 4.11
Segmentation	IMOD 4.11, EMAN 2.91
Subtomogram classification & averaging	I3 0.9.9.3, RELION 3.0.4
Density map visualization	UCSF ChimeraX, UCSF Chimera
Figure/movie creation and editing	Adobe Photoshop, Illustrator

Table S2. Cryotomogram details.

Tomogram	Fig	Dose (e/Å^2)	Pixel size (Å)	Δf (μm)	Δtilt (°)	Camera	VPP	Thickness (nm)	Image filter
20210726_09	3A	80	3.3	-1	3	K3	+	215	NAD
20201028_52	3B	80	3.3	-6	3	K3	-	110	Deconv
20210814_87	3C	80	3.3	-6	3	K3	-	110	Deconv
20191012_24	3D	80	1.75	-6	3	K3 (super-resolution)	-	170	Deconv
20201028_73	4A	80	3.3	-6	3	K3	-	170	NAD
20220108_14	4B	80	3.3	-1	3	K3	+	150	-
20201121_53	S3A	80	3.3	-1	3	K3	+	175	NAD
20210706_53	S3B	80	3.3	-1	3	K3	+	180	NAD
20210619_08	S3C	80	3.3	-1	3	K3	+	160	NAD
20210619_62	S3D	80	3.3	-1	3	K3	+	160	NAD
20210706_35	S3E	80	3.3	-1	3	K3	+	190	-
20210619_14	S3F	80	3.3	-1	3	K3	+	180	NAD
20210726_15	S4A	80	3.3	-1	3	K3	+	220	NAD
20210726_13	S4B	80	3.3	-1	3	K3	+	200	NAD
20200819_15	-	80	3.3	-6	3	K3	-	180	-
20200819_18	-	80	3.3	-6	3	K3	-	150	-
20200819_25	-	80	3.3	-6	3	K3	-	150	-
20200819_36	-	80	3.3	-6	3	K3	-	170	-
20200919_14	-	80	3.3	-6	3	K3	-	290	-
20200919_24	-	80	3.3	-6	3	K3	-	250	-
20200919_27	-	80	3.3	-6	3	K3	-	180	-
20200920_25	-	80	3.3	-6	3	K3	-	210	-
20200920_26	-	80	3.3	-6	3	K3	-	250	-
20200920_29	-	80	3.3	-6	3	K3	-	160	-
20201028_68	-	80	3.3	-6	3	K3	-	160	-
20201028_69	-	80	3.3	-6	3	K3	-	160	-
20201028_72	-	80	3.3	-6	3	K3	-	160	-
20201028_77	-	80	3.3	-6	3	K3	-	160	-
20201028_78	-	80	3.3	-6	3	K3	-	160	-
20201121_32	-	80	3.3	-1	3	K3	+	100	-
20201121_33	-	80	3.3	-1	3	K3	+	120	-
20201121_54	-	80	3.3	-1	3	K3	+	190	-
20201121_62	-	80	3.3	-1	3	K3	+	200	-

20201212_39	-	80	3.3	-1	3	K3	+	170	-
20201212_57	-	80	3.3	-1	3	K3	+	200	-
20201212_65	-	80	3.3	-1	3	K3	+	220	-
20201212_71	-	80	3.3	-1	3	K3	+	200	-
20220108_12	-	80	3.3	-1	3	K3	+	150	-
20220108_15	-	80	3.3	-1	3	K3	+	150	-
20220108_22	-	80	3.3	-1	3	K3	+	150	-
20220108_25	-	80	3.3	-1	3	K3	+	150	-
20220108_28	-	80	3.3	-1	3	K3	+	150	-
20220108_31	-	80	3.3	-1	3	K3	+	150	-

Fig = figures that include this dataset; the datasets not included in a figure were used for subtomogram averaging and 3D classification. NAD: Nonlinear Anisotropic Diffusion filter. Deconv: deconvolution filter (https://github.com/dtegunov/tom_deconv)

References

1. Simossis, V.A. & Heringa, J. PRALINE: a multiple sequence alignment toolbox that integrates homology-extended and secondary structure information. *Nucleic Acids Res* **33**, W289-294 (2005).
